# Hidden in plain sight? Identifying patient-authored publications

**DOI:** 10.1186/s40900-022-00346-w

**Published:** 2022-04-11

**Authors:** Jacqui Oliver, Dawn Lobban, Laura Dormer, Joanne Walker, Richard Stephens, Karen Woolley

**Affiliations:** 1 Envision Pharma Group, Barons Court, 3 Swan St, Wilmslow, SK9 1HF UK; 2 Future Science Group, London, UK; 3 Patient Author and Co-Editor-in-Chief of Research Involvement and Engagement, London, UK; 4 University of Queensland, Brisbane, QLD Australia; 5 University of the Sunshine Coast, Maroochydore, QLD Australia; 6 Envision Pharma Group, Sydney, Australia

**Keywords:** Authorship, Diversity, Equity and inclusion, Patient author, Patient and public involvement, Publications, PubMed

## Abstract

**Background:**

Patient advocates are increasingly authoring peer-reviewed publications that could enhance patient care and understanding of the lived experience. Although patient authorship may be seen as an innovation in the peer-reviewed publication environment and some may not be aware of or accept patient authorship, we know patient-authored publications exist. However, identifying patient-authored publications is often challenging and time-consuming.

**Main body:**

In this commentary, we propose a definition for a patient author and patient-authored publications. We outline factors driving the increase in patient authorship, including patient interest, recognition of the value of including the patient voice and major funders recognising the importance of involving patient advocates in research. Evidence and experience-based guidance on patient authorship is emerging, and we highlight practical guidance for patient advocates on authoring peer-reviewed publications. To gain a better understanding of patient authorship, an efficient method is needed to identify patient-authored publications. A dataset on patient-authored publications could be used for a range of quantitative and qualitative research studies. The affiliation search function in PubMed can provide an easy, and reproducible way to identify a dataset of patient-authored publications in the international peer-reviewed literature, but only if patient authors include a standard metatag, (e.g. Patient Author) as one of their listed affiliations, combined with other affiliations as appropriate. From 2020 to 2021, there was a nine-fold increase in patient-authored publications in PubMed identified using the Patient Author tag. We recognize that terminology can be contentious and some authors may prefer alternative metatags. Further efforts are required to gain consensus on a suitable, standard metatag or set of metatags to use to show the true extent of patient authorship.

**Conclusion:**

Patient authorship is not only legitimate, but it also exemplifies the principles of diversity, equity and inclusion. Stakeholders in the publication community need to review their policies and procedures to identify and address barriers to patient authorship. Patient advocates, funders, researchers and publishers could all help to promote awareness and acceptance of patient authorship and the merits of using a standard metatag or set of metatags, so that patient-authored publications are no longer hidden in plain sight.

## Plain English summary

Some patients are leading or helping with medical research to improve understanding of their condition and patient care. To share research findings, patients can author articles published in scientific journals. These articles are reviewed by experts and are known as peer-reviewed publications. Patient authors can provide unique and valuable insights from their experience of living with a condition. Demonstrating that patients can be authors would be easier if there was a quick way to find patient-authored publications. In this article, we describe who a patient author is and what patient-authored publications are. We identify factors that may encourage patients to author research publications. We highlight the practical guidance available to help patient authors and those working with them. To help future research about patient authorship, we need a way to find patient-authored publications. One way is for patients to include a standard search term, such as ‘Patient Author’ in the affiliation section of their publication. Like all authors, patient authors can list more than one affiliation, such as their workplace if they wish. We used the ‘Patient Author’ search term to look at publications in PubMed, a free resource to access scientific publications. We found the number of patient-authored publications using the ‘Patient Author’ tag increased nine times from 2020 and 2021. We encourage patients, funders, researchers and publishers to use a standard metatag or an agreed set of metatags. This could make it easier to find and raise awareness of patient-authored publications.

## Background

Many stakeholders now recognize the importance of involving patients in research to understand diseases and to develop medicines [1, 2]. Reflective of this change, patient advocates have started to author peer-reviewed publications from research across the medicine development life cycle [3–5]. Patient authorship may be seen as an innovation in the peer-reviewed publication environment and, as with any innovation, some stakeholders may not be aware of or be willing to accept it. We know patient-authored publications exist, but they can be difficult to find. Increased efforts are required to raise awareness and acceptance of patient-authored publications. The time has come to put them in plain sight.

In this editorial, we deliberately focus on publications authored by patients, but recognise that terminology may be contentious and that other stakeholders (e.g. Service User Author, Public Author) can and should author publications. In keeping with our intended focus, we propose a definition of patient author and patient-authored publications, identify factors driving an increase in patient authorship, highlight emerging evidence and guidance on patient authorship and recommend actions that could be taken to accelerate research, metrics, awareness and acceptance of patient authorship. We hope that this article stimulates respectful discussions, from a broad and diverse community, about patient authorship and may lead to consensus on a suitable standard metatag or, indeed, a standard set of metatags to identify patient-authored publications.

## Definition of a patient author and patient-authored publications

Currently, there are no standard definitions of a patient author or a patient-authored publication, so we propose the definitions below.

A *patient author* should meet all of the following criteria:

- A person who lives with or is affected by a disease or condition (i.e. a broad definition of patient that includes those with lived conditions or receiving health or social care, caregivers, family members and members of patient advocacy groups who represent them) [6, 7].
- A person who provides unique and valuable input from the patient perspective to the publication.
- A person who meets all the criteria required for authorship (e.g. criteria from the International Committee of Medical Journal Editors [ICMJE]) [8].

A *patient-authored publication* is an article authored or co-authored by a patient and is published in a peer-reviewed journal.

To meet the high standards of authorship, patient authors should fulfill all the ICMJE criteria, including contributing to the research and providing input when developing the manuscript. With valued input from patient advocates, we have ‘translated’ the ICMJE criteria, providing examples of the types of contributions patient advocates can make to meet each criterion [9]. If author criteria were adjusted for patient authors, as has been recently considered [10], there is a risk that this could, inadvertently, lead to tokenistic or guest authorship rather than supporting genuine equality of status between patient and academic/expert contributions in research publications.

## Factors driving the increase in patient authorship

Patient authorship is expected to increase as a result of several factors, including:

- *Patient interest* Patients want to make a difference and to contribute to research that will have an impact on patient care. As learning and experience among patient advocates have increased, more patients have seen opportunities for increasing their involvement in research publications.
- *Recognition by major funders* Funders such as the Patient-Centered Outcomes Research Institute (PCORI) in the United States [11], the National Institute of Health Research (NIHR) in the UK, the National Health and Medical Research Council (NHMRC) in Australia and the Japan Agency for Medical Research and Development (AMED) explicitly require or strongly encourage patient involvement in research and some proactively support patient authorship [12–15].
- *Including the patient voice* There is increased recognition across the scientific community that the patient voice brings unique insights to the understanding of the lived experience and should be included in peer-reviewed publications.
- *Patient research* Patients are leading or partnering with others (e.g. academic or industry partners) to conduct research and have recognized the need to share their findings by authoring publications in peer-reviewed publications [16, 17]. Anecdotally, it seems that the opportunities for patients to author publications may have increased during the COVID-19 pandemic. The ability to collaborate remotely has eased the financial, operational and health burdens that can be encountered when patient advocates have had to travel to attend face-to-face research and authorship meetings.
- *Patient involvement at congresses and journals* Increasingly congresses and journals are supporting patient involvement in presenting, publishing and peer-reviewing research [18–20].
- *Patient publication steering committees* Industry innovators are establishing patient publication steering committees to provide the oversight necessary for patients to author or co-author timely and high-quality publications that address unmet needs, as identified by patients [21].
- *Plain language summaries (PLS)* There is growing interest in PLS of peer-reviewed research publications [22], which has opened doors for patient authors through increased understanding and opportunities to develop authorship skills through co-creating PLS [23].

## Emerging evidence and guidance on patient authorship

**Fig. 1:**
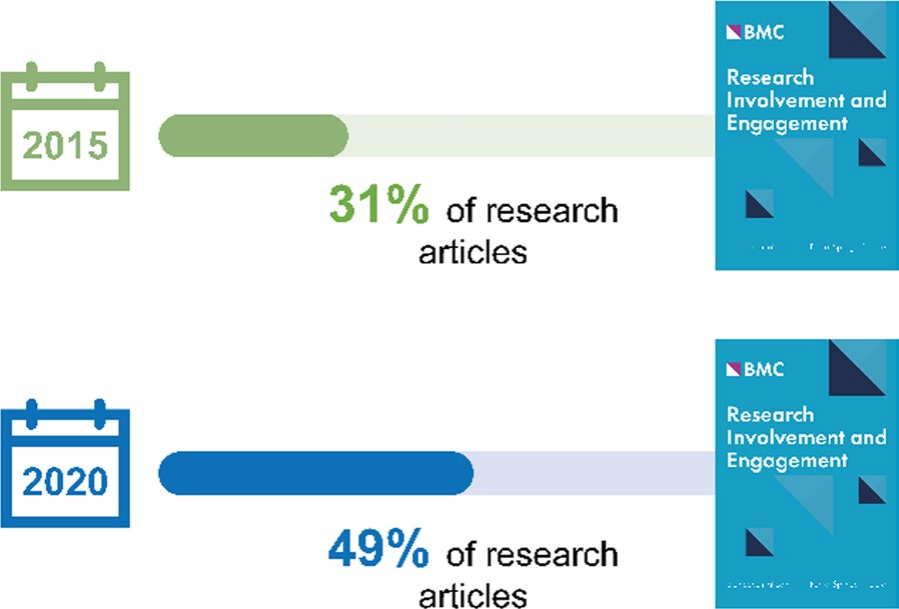
Increase in patient-authored publications in *Research Involvement and Engagement* from 2015 to 2020*. *Patient-authored publications were identified by hand and publications involved at least one patient author

Despite the challenges of identifying patient-authored publications and, by extension, the lessons that might be gained from them, researchers have started to publish evidence and provide initial guidance on patient authorship. Based on handsearching methods, the number of patient-authored publications appears to be increasing and may vary across journals. In *The BMJ*, a general medical journal that has pioneered patient partnership strategies, the number of patient-authored publications in 2015–2016 was very low (1.9%; 1 of 52 research articles) [24]. In *Research Involvement and Engagement,* which has a strong focus on patient involvement in research and was the first journal to have a patient advocate as Co-Editor-in-Chief, the number of patient-authored publications increased from 31% (4 of 13 research articles) in 2015 to 49% (34 of 69 research articles) in 2020 (Fig. 1) [25]. A recent survey of medical journal editors has shown that most editors agree that patients can be authors and contribute positively to research [10].

Evidence-based and experience-based guidance on patient authorship is also emerging. A systematic review, co-authored with patients, on patient involvement in research publications, identified 21 evidence-based recommendations to help minimize the potential risks and maximize the potential benefits of patient authorship [26]. Patient partners and researchers from the Chronic Pain Network have published a narrative review that provides guidance for patient and nonpatient authors on patient authorship [27]. More recently, based on demand by patient advocates for practical guidance on how to author peer-reviewed publications, international patient advocates and certified medical publication professionals created the first free online ‘Patients in Publications’ training course for patient advocates (launched in June 2021) [28, 29].

## Recommendations to accelerate research, metrics, awareness and acceptance of patient authorship

To gain a better understanding of patient authorship and to counter critics who question whether patients can be authors, research is required. However, a major challenge for conducting this research is the difficulty in identifying patient-authored publications. There has not been a clear or consistent way of identifying patient authors [26, 30]. Searching articles by hand for ‘clues’ on which authors may be patients (e.g. via their affiliation, disclosures, contribution statements in the article or conducting internet searches on listed authors) is time-consuming and may underestimate the number of patient-authored publications, as well as limiting the scope of research that should be undertaken. Some researchers have made the effort to contact lead authors directly to ask whether any patients were co-authors [31]. While this method should provide definitive answers, it would also be time-consuming, especially for large-scale studies. If there was an effective way to search for patient-authored publications, then publication datasets could be generated that could facilitate a wide range of quantitative and qualitative research studies on patient authorship. In addition, the number and type of patient-authored publications of patient-led or patient-partnered research could be used, as part of a panel of metrics, to help different stakeholders (e.g. patient advocacy groups, research sponsors, grant reviewers) encourage and evaluate patient involvement in research.

Based on our pilot test, we propose that PubMed could be used as an effective way to search the international peer-reviewed literature for patient-authored publications [25]. Specifically, the affiliation search function in PubMed could provide an easy, quick, free and reproducible way to generate a dataset of patient-authored publications. However, the success of this searching method does rely on the inclusion of ‘Patient Author’ (or another standard metatag) as one of a patient author’s affiliations. The use of the specific term ‘Patient Author’ quickly and clearly reinforces to the reader (and before that, to the editor and peer-reviewers) that the patient’s contribution warranted authorship. Patient advocates are now being advised to include ‘Patient Author’ in their affiliations [28], and some publishers and researchers have started to raise awareness of its use [32]. Researchers have recently called for the Guideline for Reporting Involvement of Patients and the Public (GRIPP2) reporting checklist to include a section to state whether the research included patient and public co-authors [31].

**Fig. 2:**
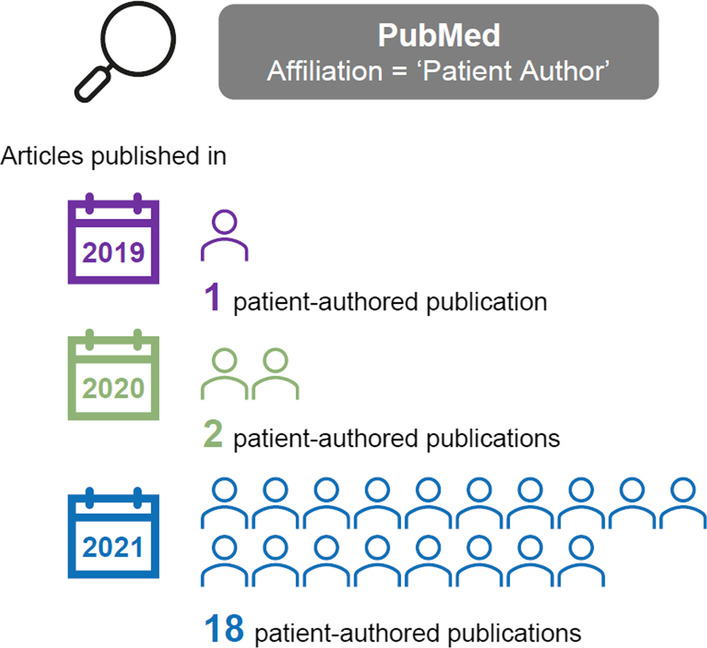
Increase in publications using ‘Patient Author’ as an affiliation tag in PubMed from 2019 to 2021*. *Publications involved at least one patient author. The search includes PubMed records with a publication date between 1 January 2019 and 31 December 2021

Between 2020 and 2021, we found that the number of publications retrieved by using ‘Patient Author’ in the PubMed affiliation search function, while still limited, has increased nine-fold (Fig. 2) [25]. Notably, the dataset of retrieved patient-authored publications included those where patients were the lead or sole author (13 publications). This reinforces that patient authorship is not and should not be tokenistic. Patient authors can clearly collaborate with co-authors, but they do not have to depend on co-authors to publish. We caution that datasets generated by the ‘Patient Author’ affiliation search function would not currently reflect the true extent of patient-authored publications, as further efforts are required to gain consensus and standardization of a suitable metatag or set of metatags. We recognize and respect that patients may have other affiliations, such as academic roles, or may prefer to use alternative descriptions for a patient representative (e.g. Patient Partner, Patient Advocate, Caregiver). These affiliations can be included in addition to ‘Patient Author’, in the same way that other authors may include multiple affiliations. The Boolean features available through the PubMed search function would allow for a standard set of metatags to be used. An extensive list of metatags, however, could prove cumbersome.

In terms of raising awareness and acceptance of patient authorship, all those involved in the peer-reviewed publication community (e.g. funders, researchers, editors, peer-reviewers, publishers, publishing organizations) need to recognize that patients can be and indeed already are authors. Peer-reviewed literature should include the voice of patients, as they are the experts in what it is to like to live with a condition. On a practical level, stakeholders in the publication community should review their policies and procedures to identify and address barriers to patient authorship. For example, a recent survey highlighted that only 3.6% of editors-in-chief (4 of 110) had a policy that specifies how patients or patient partners should be considered as authors [10]. Addressing this gap among the guardians of the peer-reviewed literature could help facilitate greater awareness and acceptance of patient authorship. Patient advocates and their collaborators (e.g. industry, nonindustry sponsors) can also encourage patients who are or who want to be authors to undertake a free training course on how to publish and how to use the ‘Patient Author’ affiliation metatag [28]. Improving their knowledge of the publication process could help patient authors address some of the power imbalances that may occur in co-authorship teams [32]. We also recommend reflective use of authorship experience tools to allow patient and nonpatient authors to consider the challenges and benefits of co-authoring publications [26]. These tools have been co-created with patient leaders and are based on a robust, evidence-based, patient involvement framework [26]. Social media platforms can also be used to raise awareness and acceptance of patient authorship. For example, the use of #PatientAuthor on Twitter and the ‘Patients in Publications’ group on LinkedIn [33] are being used to capture ideas, share experiences and provide examples of patient-authored publications. Notably, use of a specific term like ‘Patient Author’ on social media helps retrieve information related to patient-authored publications. Use of broader terms (e.g. ‘Patient Partner’, ‘Patient Participant’) retrieves information that may or may not be related to patient-authored publications.

## Conclusions

Patient-authored publications need to be in plain sight. Patient advocates, funders, researchers and publishers could all help to promote awareness and acceptance of patient-authored publications. We hope this article serves as a catalyst for respectful discussions, from a broad and diverse community, about patient authorship. On a practical level, we hope one outcome would be reaching consensus on a suitable standard metatag or, indeed, a standard set of metatags to reveal the true extent of patient authorship. Datasets of patient-authored publications could be used in a range of quantitative and qualitative research studies to improve our understanding of patient authorship and to encourage more patients to become authors and contribute their unique and valuable perspectives to the peer-reviewed literature.

“Of course, patients should be involved as co-authors of medical research papers.

It’s our story you’re telling.”

Richard Stephens

## Data Availability

Not applicable.

## Abbreviations

AMED: Japan Agency for Medical Research and Development
GRIPP2: Guideline for reporting involvement of patients and the public
ICMJE: International Committee of Medical Journal Editors
NIHR: National Institute of Health Research
NHMRC: National Health and Medical Research Council
PCORI: Patient-Centered Outcomes Research Institute
PLS: Plain language summaries
